# Sensory discrimination between innocuous and noxious cold by TRPM8-expressing DRG neurons of rats

**DOI:** 10.1186/1744-8069-8-79

**Published:** 2012-10-24

**Authors:** Ignacio Sarria, Jennifer Ling, Guang-Yin Xu, Jianguo G Gu

**Affiliations:** 1 Department of Anesthesiology and the Graduate Program in Neuroscience, The University of Cincinnati College of Medicine, PO Box 670531, 231 Albert Sabin Way, Cincinnati, OH, 45267, USA; 2 Institute of Neuroscience, Department of Neurobiology, Key Laboratory of Pain Basic Research and Clinic Therapy, Soochow University, Suzhou, 215123, PR China

## Abstract

The TRPM8 channel is a principal cold transducer that is expressed on some primary afferents of the somatic and cranial sensory systems. However, it is uncertain whether TRPM8-expressing afferent neurons have the ability to convey innocuous and noxious cold stimuli with sensory discrimination between the two sub-modalities. Using rat dorsal root ganglion (DRG) neurons and the patch-clamp recording technique, we characterized membrane and action potential properties of TRPM8-expressing DRG neurons at 24°C and 10°C. TRPM8-expressing neurons could be classified into TTX-sensitive (TTXs/TRPM8) and TTX-resistant (TTXr/TRPM8) subtypes based on the sensitivity to tetrodotoxin (TTX) block of their action potentials. These two subtypes of cold-sensing cells displayed different membrane and action potential properties. Voltage-activated inward Na^+^ currents were highly susceptible to cooling temperature and abolished by ~95% at 10°C in TTXs/TRPM8 DRG neurons, but remained substantially large at 10°C in TTXr/TRPM8 cells. In both TTXs/TRPM8 and TTXr/TRPM8 cells, voltage-activated outward K^+^ currents were substantially inhibited at 10°C, and the cooling-sensitive outward currents resembled A-type K^+^ currents. TTXs/TRPM8 neurons and TTXr/TRPM8 neurons were shown to fire action potentials at innocuous and noxious cold temperatures respectively, demonstrating sensory discrimination between innocuous and noxious cold by the two subpopulations of cold-sensing DRG neurons. The effects of cooling temperatures on voltage-gated Na^+^ channels and A-type K^+^ currents are likely to be contributing factors to sensory discrimination of cold by TTXs/TRPM8 and TTXr/TRPM8 afferent neurons.

## Introduction

Sensory discrimination between innocuous and noxious cold is essential for humans and animals to survive in nature because painful cold sensations can warn an individual to avoid prolonged exposures to harmfully low temperatures. Psychophysical studies in normal humans show that cooling temperatures in the range of 30 to 15°C are innocuous, while temperatures below 15°C provokes painful sensations that are often described as multiple modalities, including burning, stinging, tingling, and pressing [1]. Operant behavioral tests in rats reveal innocuous and noxious cooling temperatures [2] that are in agreement with human psychophysical tests. The sensory discrimination between innocuous and noxious cold can be altered under many conditions, including diseases. For example, under pathological conditions such as complex regional pain syndrome, innocuous cold can induce painful sensations that are clinically called cold allodynia [3]. Cold allodynia appears to have no positive physiological meaning and is a clinical problem that requires medical management. Unfortunately, cold allodynia cannot be effectively treated at the present time partially because the sensory discrimination between innocuous and noxious has not been well understood.

Important progress has been made during last 10 years in understanding the transduction of cold stimulation by primary afferents. TRPM8 was discovered to be a principal transducer for cold stimuli in primary afferents [4-9]. Other molecules such as TRPA1 [10] and TREK1 channels [11,12] are also proposed to be candidates of cold transducers. It has been thought that sensory discrimination between innocuous and noxious cold might be at a transducer level, with one type of transducer molecules (e.g. TRPM8) for innocuous cold and another type (e.g. TRPA1) for noxious cold. However, this hypothesis has been challenged by factors including the thermal sensitivity and expression patterns of TRPM8 channels. For example, TRPM8 can be activated progressively by cooling temperatures between 28°C to 10°C, covering both innocuous and noxious cold temperatures [7,8]. TRPM8 channels are found to be expressed in both non-nociceptive and nociceptive sensory neurons [13-16]. The thermal sensitivity and expression patterns of TRPM8 suggest that this single cold transducer can be involved in both innocuous cooling and noxious cold transduction. However, this generates a puzzle as how innocuous and noxious cold stimuli can be discriminated by primary afferents if a single cold transducer is used for detecting both innocuous and noxious cold stimuli. To answer this question, one has to first know if TRPM8-expressing non-nociceptive and TRPM8-expressing nociceptive afferents indeed differentially respond to innocuous and noxious cold stimuli, respectively.

Many previous studies have focused on the transduction function of TRPM8 channels in cold-sensing afferent neurons. Attention has not been well paid to the action potential firing properties of TRPM8-expressing neurons, an issue that is important for understanding sensory discrimination between innocuous and noxious cold. It is important because action potentials are the final read out of sensory response to cold stimuli. Sensory discrimination between innocuous and noxious cold should be reflected by the ability of non-nociceptive and nociceptive afferent neurons to generate sensory impulses at innocuous and noxious cold temperatures, respectively. Using cold-insensitive cells, we have recently shown that cooling temperatures affect action potential firing in TTXs and TTXr DRG neurons and that the effects were associated with the differential inhibition of TTXs and TTXr voltage-activated Na^+^ channels [17]. In the same study we also have shown that cooling temperatures inhibit A-type K^+^ channels, which partially contributes to the differential effects of cooling temperatures on action potential firing in TTXs and TTXr cells [17]. In another study by a different group, it has also been shown that TTXs and TTXr channels are differentially inhibited by cooling temperatures, and that TTXr channels are essential for pain at low temperatures [18]. As an extension of our previous study in cold-insensitive neurons, in the present work, we used cold-sensitive cells that expressed TRPM8 channels so we could study their membrane and action firing properties at both neutral and cooling temperatures. We also examined effects of cooling temperatures on voltage-activated inward currents and outward currents in the TRPM8-expressing neurons. Our results indicate that TTXs/TRPM8-expressing (non-nociceptive-like) and TTXr/TRPM8-expressing (nociceptive-like) afferents indeed differentially respond to innocuous and noxious cold stimuli, respectively.

## Materials and methods

### DRG neuron preparations

Adult Sprague Dawley rats (100–250 g, both genders) were used. Animal care and use conformed to National Institutes of Health guidelines for care and use of experimental animals. Experimental protocols were approved by the University of Cincinnati Institutional Animal Care and Use Committee. DRG neuron cultures were prepared as described previously [19]. In brief, rats were deeply anesthetized with isoflurane (Henry Schein, NY) and sacrificed by decapitation. DRGs were rapidly dissected out bilaterally in Leibovitz-15 medium (Mediatech Inc. VA) and incubated for 1 hour at 37°C in minimum essential medium for suspension culture (S-MEM) (Invitrogen, Grand Island, NY) with 0.2% collagenase and 0.5% dispase and then triturated to dissociate neurons. The dissociated DRG neurons were then plated on glass coverslips pre-coated with poly-D-lysine (PDL, 12.5 μg/ml in distilled H_2_O) and laminin (20 μg/ml in Hank’s Buffered Salt Solution HBSS, BD bio-science), and maintained in MEM culture medium (Invitrogen) that also contained 5% heat-inactivated horse serum (JRH Biosciences, Lenexa, KS), uridine/5-fluoro-2^′^-deoxyuridine (10 μM), 8 mg/ml glucose, and 1% vitamin solution (Invitrogen). The cultures were maintained in an incubator at 37°C with a humidified atmosphere of 95% air and 5% CO_2_. Cells were used between 24 to 72 hours after plating.

### Electrophysiology

Coverslips with cultured neurons were placed in a 0.5-ml microchamber, mounted on an Olympus IX70 inverted microscope (Olympus, USA), and continuously perfused with a bath solution (see below) at 2 ml/min. Cells were first tested with 100 μM menthol and cold bath (15°C) to pre-identify menthol/cold-sensitive cells by using the Ca^2+^ imaging method which we have previously described [20]. Patch-clamp recordings were then performed on menthol/cold-sensitive cells to study their membrane excitability and action potential firing properties under current clamp configuration, while voltage-gated Na^+^ channels and voltage-gated K^+^ channels were studied under voltage-clamp configuration. Patch-clamp electrode internal solution contained (in mM) 135 K-gluconate, 5 KCl, 2.4 MgCl_2_, 0.5 CaCl_2_, 20 BAPTA, 10.0 HEPES, 5.0 Na_2_ATP, 0.33 GTP-Tris salt, pH was adjusted to 7.35 with NaOH and osmolarity was adjusted with sucrose to 320 mOsm [21]. Recording electrode resistance after filling internal solutions (see below) was 4–6 MΩ. Junction potentials between bath and electrode solutions were calculated and corrected for in the data analysis. Current and voltage signals were recorded with an Axopatch 200B amplifier (Axon Instruments) and signals were sampled at 10 kHz and filtered at 2 kHz using pCLAMP 9.0 (Axon Instruments). Unless otherwise indicated, cells were continuously perfused with a normal bath containing (in mM) 150 NaCl, 5 KCl, 2 MgCl_2_, 2 CaCl_2_, 10 glucose, 10 HEPES, pH 7.3, osmolarity 330 mOsm, at room temperature of 24°C. In all experiments, cells that had a resting membrane potential more positive than −45 mV or a passive leak current more than 100 pA were discarded. Cells with an increase in passive leak current during cooling were also discarded.

Each menthol/cold-sensitive cell was tested with TTX (500 nM) to determine if a cell predominantly had TTXs Na^+^ currents (TTXs cell) or had TTXr Na^+^ currents (TTXr cell) as well. This was achieved under voltage-clamp configuration by recording inward currents in response to a series of voltage steps (10 mV each step, ranging from −90 to 40 mV) from a holding potential of −70 mV in the absence and presence of presence 500 nM TTX. To estimate the effects of cooling temperatures on voltage-gated Na^+^ channels and voltage-gated K^+^ channels, whole-cell inward currents and outward currents were evoked by a series of voltage steps (10 mV each step, ranging from −90 to 40 mV) from a holding potential of −70 mV, and the experiments were performed at 24°C and 10°C. Isolation of sodium currents and potassium currents were not performed in this study because we intended to keep these menthol/cold-sensitive cells in a relatively normal condition for the experiments described below. To determine the effect of cooling temperatures on action potential firing properties in TTXs and TTXr cells, a cold ramp was used to drive depolarization; membrane potentials and action potential firing were continuously recorded while temperature of perfusion bath solution dropped from 29 to 10°C or to the temperature at which action potentials were completely failed. The cooling temperature was controlled to have a slow ramp that took 3 min from 29 to 10°C.

### Application of cooling temperatures and drugs

The temperatures of bath solutions were controlled by a Peltier cooling device (Model TCM-1, Warner Instrument, CT, USA), which were delivered to patched cells from a short tube (0.2 cm L, 500 μm ID) with the outlet 500 μm away from the recorded cells. The temperatures at the recording sites were continuously recorded with a thermal probe that attached to the controller of the Peltier cooling device. Drugs were delivered by another short tube like the one described, but without temperature control; thus they were delivered and tested at room temperature of ~24°C. For voltage- and current-clamp experiments, a targeted temperature of 24°C or 10°C was reached first and then voltage and/or current protocols were applied to determine changes in currents and membrane properties, respectively.

### Data analysis

Whole-cell recordings from voltage and current clamp experiments were analyzed using Clampfit 9 software. The measurements of membrane and action potential parameters were performed in the same manner as a previous study by us [17]. Data are reported as mean ± SEM. Statistical significance (p < 0.05) was assessed by Student’s *t* test.

## Results

**Figure 1:**
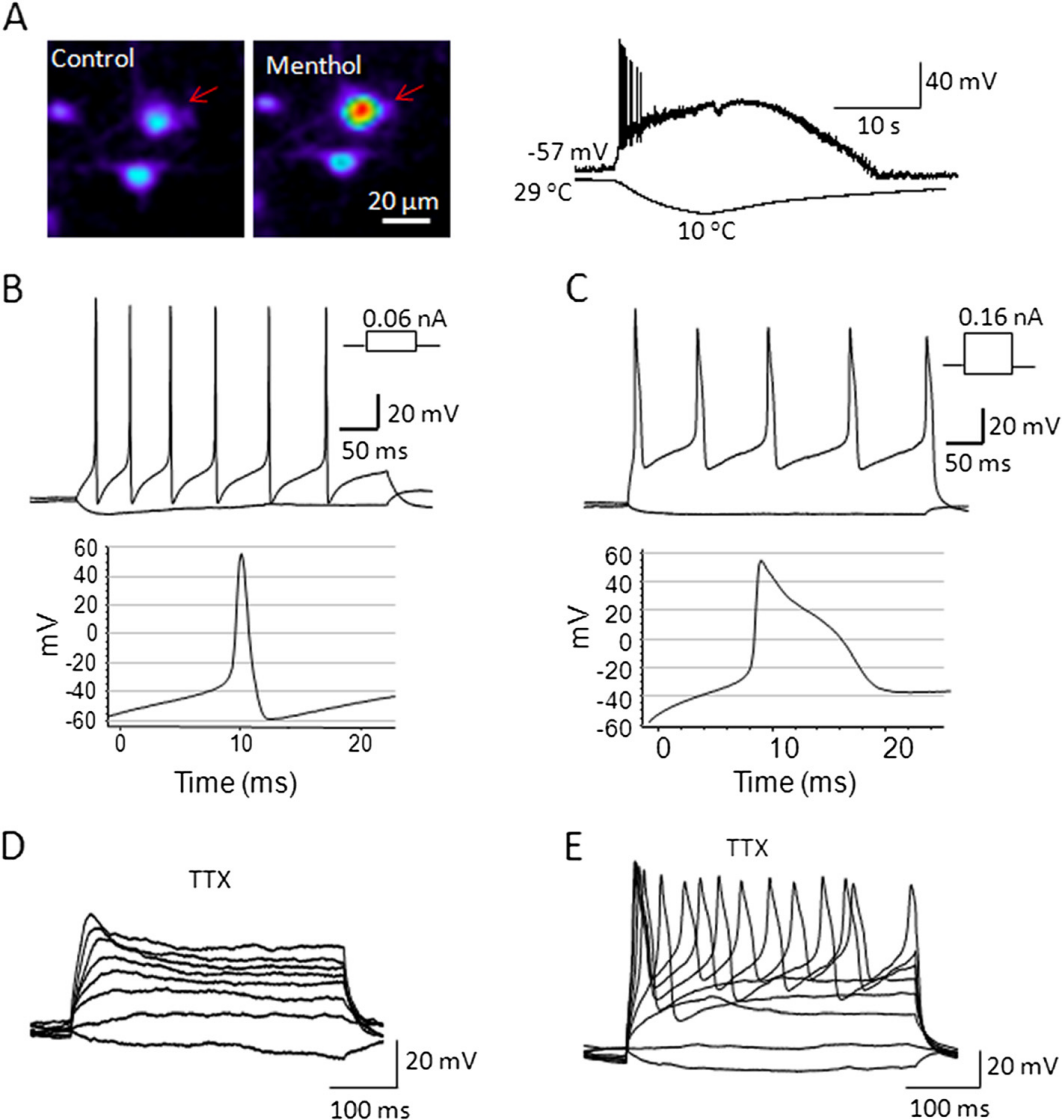
**Membrane and action potential properties of TTXs/TRPM8 and TTXr/TRPM8 DRG neurons. A**) A menthol-sensitive DRG neuron (arrow indicated) identified by the Ca^2+^-imaging method (left and middle panels). Under current-clamp configuration, a menthol-sensitive cell responded to cold stimulation with membrane depolarization and action potential firing (Right panel). Membrane response was shown in the upper trace and temperature drop in the lower trace. **B-C**) Action potential firing in response to current injection through patch-clamp electrode in two menthol/cold-sensitive cells. One cell had lower threshold for action potential firing and narrow action potentials (**B**), and the other showed higher threshold and broader action potentials (**C**). **D-E**) Effects of TTX (500 nM) on action potential firing in menthol/cold-sensitive cells. For the cell type represented in B, action potential firing was sensitive to TTX and abolished in the presence of 500 nM TTX (TTXs/TRPM8 cell, D). For the cell type represented in C, action potential firing is resistant to TTX block (TTXr/TRPM8 cell, E). Except the right trace in A, all experiments were performed at 24°C. Membrane parameters and statistical comparison are summarized in Table 1.

We used the calcium imaging method with the TRPM8 agonist menthol (100 μM) to pre-identify cells that likely expressed TRPM8 channels (Figure 1A left and middle panels) [20]. These menthol-sensitive cells were then patch-clamp recorded. Similar to our previous studies [21], cooling temperatures could evoke inward currents, resulting in membrane depolarization and action potential firing in these menthol/cold-sensitive cells (Figure 1A right panels). These cells are therefore TRPM8-expressing cold-sensing cells [21]. At 24°C, we examined action potential firing properties of these TRPM8-expressing cells in response to the injection of depolarizing currents through patch-clamp electrodes (Figure 1B and C). Two subtypes of TRPM8-expressing cells could be identified based on the shapes of their action potentials (Figure 1B). One subtype had narrow action potentials (Figure 1B left), and the action potentials were TTX sensitive (TTXs) and completely abolished by TTX (500 nM) (Figure 1D). Accordingly, in this study this subtype of cells was called TTXs/TRPM8. The other subtype of cells fired broader action potentials and each action potential displayed a hump at its repolarization phase (Figure 1B right). These cells remained firing action potentials in the presence of TTX (500 nM) and thus were termed TTXr/TRPM8 in this study (Figure 1E). In addition to the action potential width, TTXr/TRPM8 cells had a significantly higher threshold for action potential firing and larger afterhyperpolarization (AHP) amplitude (Table 1) than TTXs/TRPM8 cells. Other membrane parameters including resting membrane potentials, membrane input resistance, and AHP peak values were not found to be significantly different between the two subtypes of TRPM8-expressing cells (Table 1).

**Table 1:** Membrane parameters of TTXs/TRPM8 and TTXr/TRPM8 DRG neurons

**Cell type**	**RMP (mV)**	**Threshold (mV)**	**AP width (W**_**1/2,**_**ms)**	**AHP amplitude (mV)**	**AHP peak (mV)**	**Input R (MΩ)**
TTXs/TRPM8 n = 12	−51.7 ± 0.7	−30.9 ± 1.4†	3.5 ± 0.2†	15.4 ±1.0†	−46.2 ± 1.1	437 ± 71
TTXr/TRPM8 n = 11	−51.0 ± 1.3	−19.3 ± 2.0	4.9 ± 0.4	25.8 ± 1.5	−45.1 ± 1.4	535 ± 35

Cells were pre-identified by the Ca^2+^ imaging method for their cold- and menthol-sensitivity and by voltage-clamp recording for the sensitivity of voltage-activated Na^+^ channel currents to 500 nM TTX. TTXs/TRPM8: TTXs cells that are sensitive to menthol and cold; TTXr/TRPM8: TTXr cells that are sensitive to menthol- and cold. Cells were then under current-clamp configuration and experiments were performed at 24°C. RMP: resting membrane potential; AP: action potential; AHP: afterhyperpolarization; R: resistant. Data represent mean ± SEM. † P < 0.05, TTXs/TRPM8 vs. TTXr/TRPM8.

**Figure 2:**
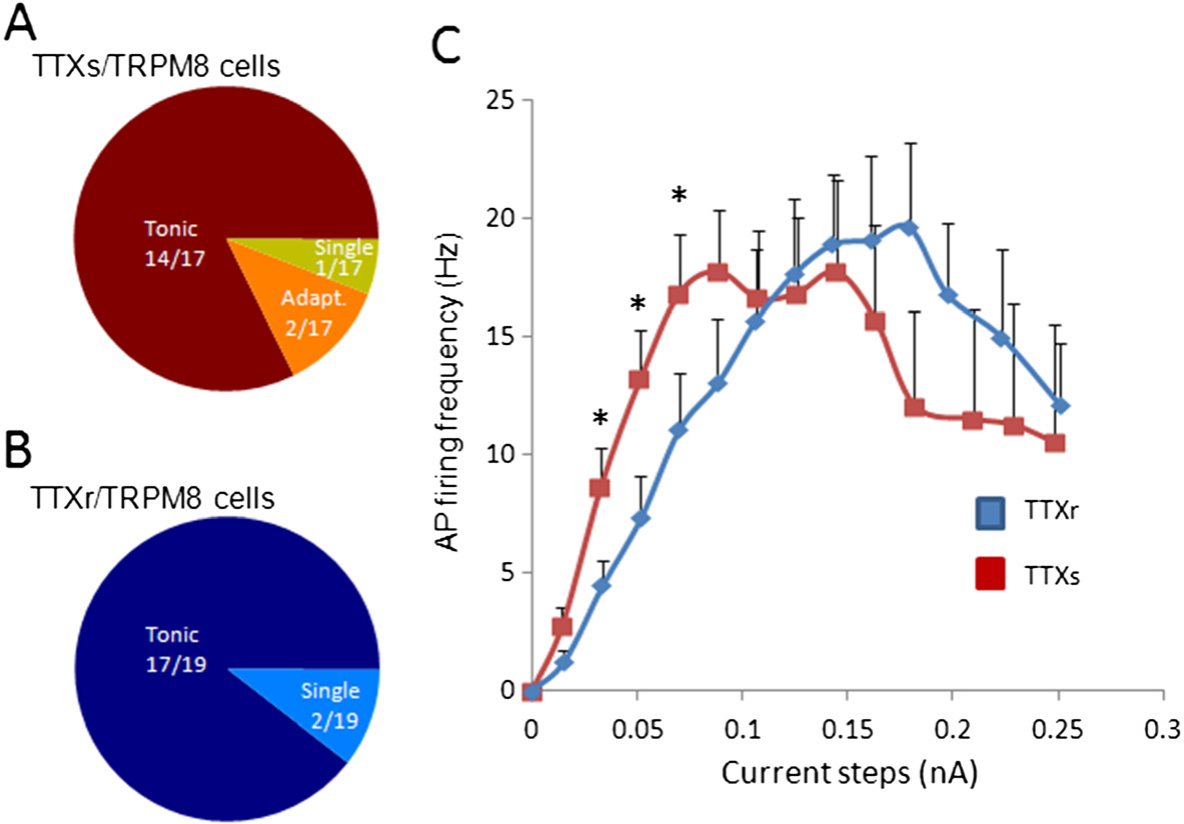
**Action potential firing patterns and frequency of TTXs/TRPM8 and TTXr/TRPM8 DRG neurons. A-B**) Fraction of cells that had tonic or other types of action potential firing in TTXs/TRPM8 (**A**) or TTXr TRPM8 cells (**B**). **C**) Relationship between action potential firing frequency and depolarizing current steps for the cells that fired tonic action potentials. Red, TTXs/TRPM8 cells. Blue, TTXr/TRPM8 cells. Data represent mean ± SEM, *P < 0.05.

The majority of TTXs/TRPM8 neurons (14/17 cells) and TTXr/TRPM8 neurons (17/19 cells) fired action potentials tonically in response to maintained depolarization (Figures 1B and C; 2A and B). Only a small number of TRPM8 cells showed other action potential firing patterns such as single or adaptive firing (Figure 2A and B). For both subtypes of TRPM8-expressing cells displaying tonic firing pattern, action potential frequency increased with increasing depolarizing currents that were injected to the cells (Figure 2C). After reaching the peak, action potential firing frequency tended to reduce when depolarizing currents were further increased. In comparison with TTXs/TRPM8 cells, TTXr/TRPM8 cells fired action potentials at a relatively lower frequency before peak frequency was reached. For example, at the depolarizing step of 0.05 nA, action potential frequency was 7.32 ± 0.05 Hz (n = 14) for TTXr/TRPM8 cells, significantly lower than that of TTXs/TRPM8 cells (13.18 ± 0.05 Hz, n = 11, Figure 2C).

**Figure 3:**
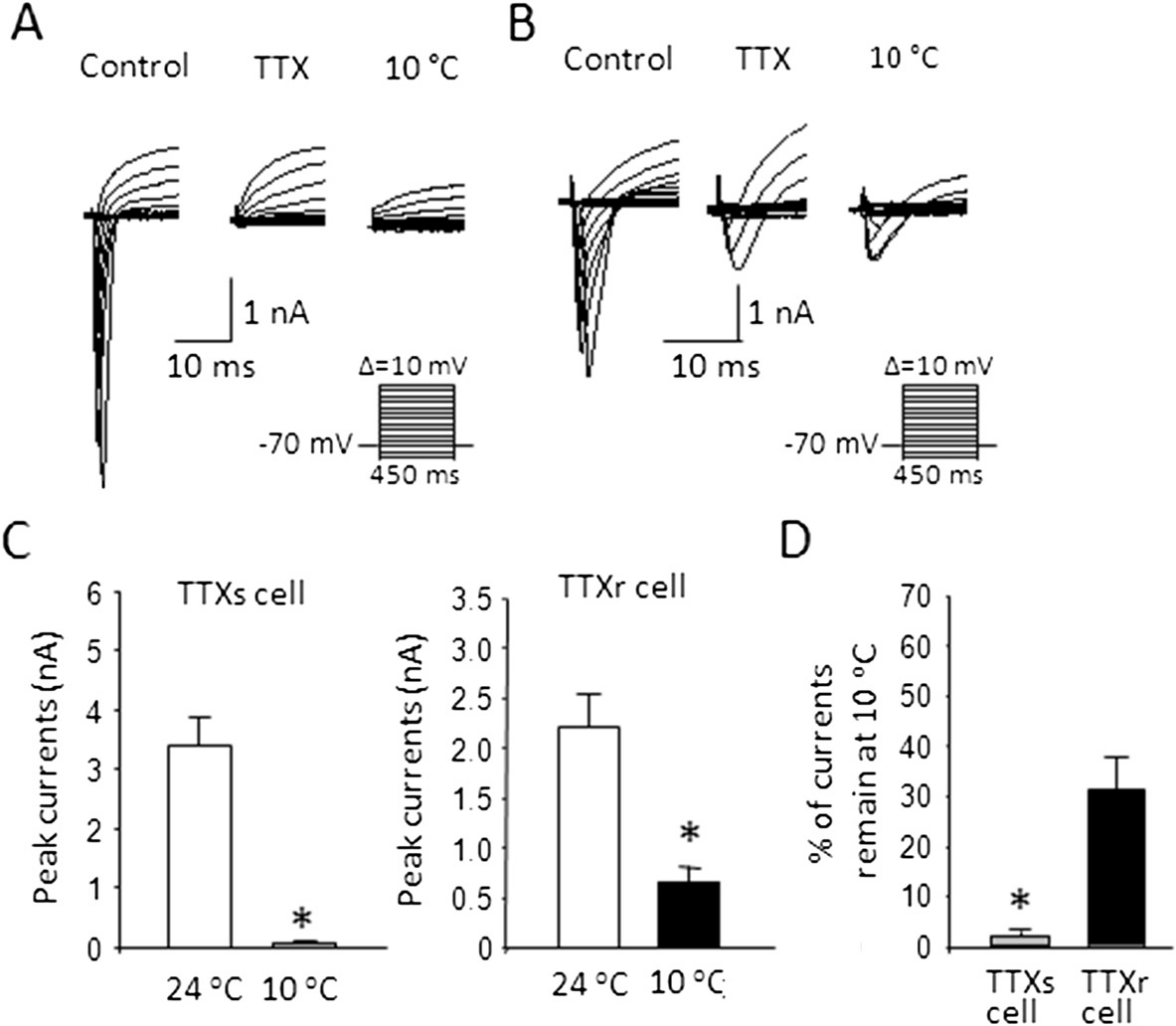
**Inhibition by cooling temperatures of voltage-activated inward currents in TTXs/TRPM8 and TTXr/TRPM8 DRG neurons. A**) Sample traces of voltage-activated inward currents recorded from a TTXs/TRPM8 cell at 24°C (left), in the presence of 500 nM TTX (middle), and at 10°C (Right). **B**) Experiment was similar to (A) except the recorded cell was a TTXr/TRPM8 neuron. **C**) Summary of peak amplitude of the inward currents recorded at 24°C and 10°C. Left panel: TTXs/TRPM8 cells. Right panel: TTXr/TRPM8 cells. **D**) Remaining inward currents at 10°C. The remaining inward currents at 10°C are expressed as the percentage of peak inward currents at 24°C. Data represent mean ± SEM, *P < 0.05.

Under voltage-clamp configuration and at 24°C, giving voltage steps to both TTXs/TRPM8 and TTXr/TRPM8 cells elicited inward currents followed by outward currents (Figure 3A left panel and 3B left panel). For TTXs/TRPM8 cells, inward currents could be completely inhibited in the presence of TTX (500 nM), indicating that the inward currents were mediated entirely by TTX-sensitive Na^+^ channels (Figure 3A middle panel). The inward currents of the TTXs/TRPM8 cells were also almost completely abolished by the cooling temperature of 10°C (Figure 3A right panel, 3C). For example, peak inward currents reached 3.32 ± 0.54 nA at 24°C, but were reduced to 0.08 ± 0.03 nA (n = 6, Figure 3C left panel) or 2.3 ± 0.9% (n = 6, Figure 3D) at 10°C. For TTXr/TRPM8 cells, inward currents could only be partially blocked by TTX (500 nM, Figure 3B left and middle panels), indicating that both TTX-sensitive and TTX-resistant Na^+^ channels were involved in the inward currents in this subtype of TRPM8-expressing cells. Different from TTXs/TRPM8 cells, the inward currents of TTXr/TRPM8 cells remained substantially large at 10°C (Figure 3B right). For example, peak inward currents were 2.22 ± 0.31 nA (n = 6) at 24°C, and were 0.66 ± 0.15 nA (n = 6, Figure 3C right panel) or 31.6±6.4% (n = 6, Figure 3D) at 10°C.

**Figure 4:**
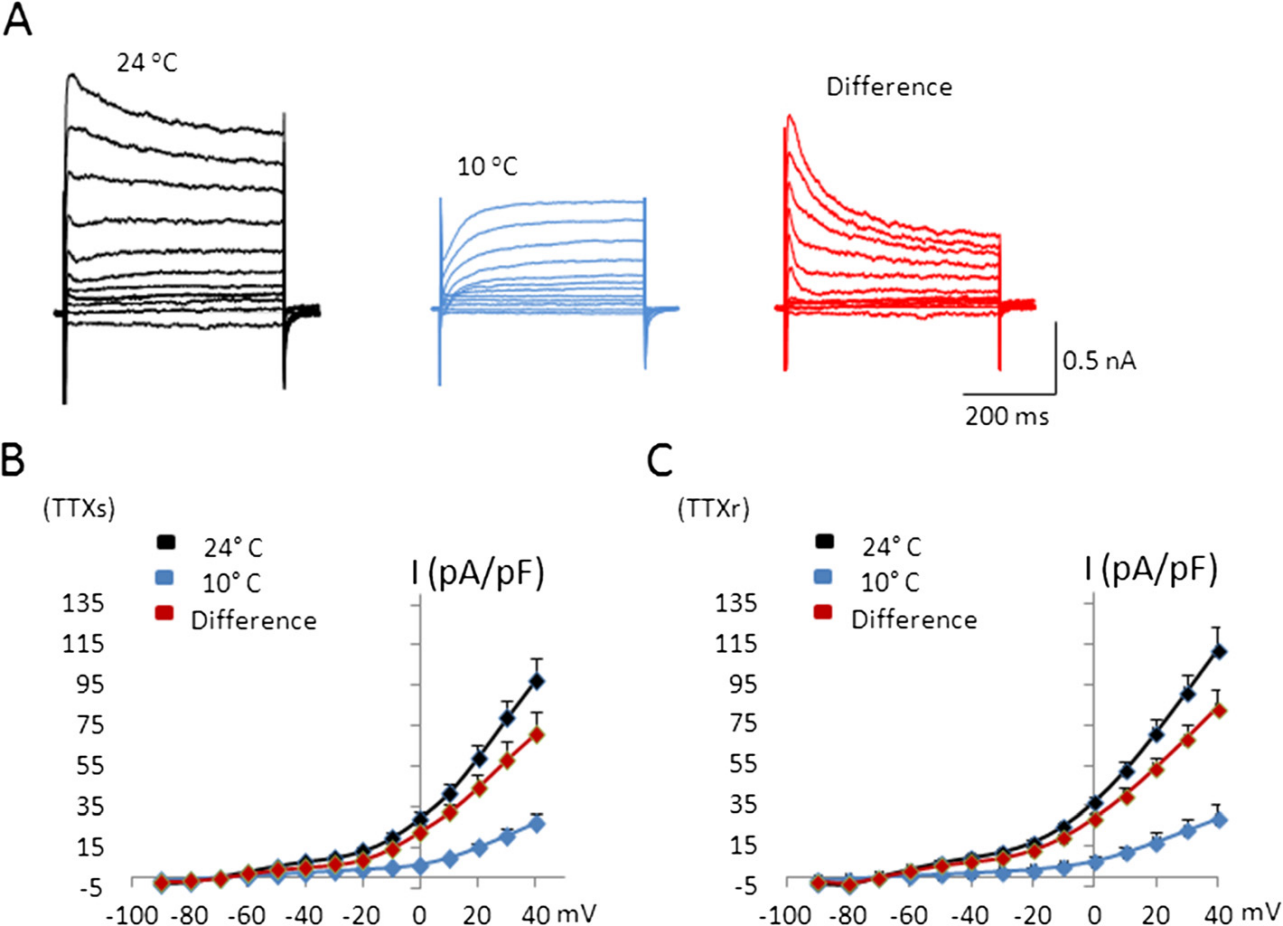
**Inhibition by cooling temperatures of voltage-activated outward currents in TTXs/TRPM8 and TTXr/TRPM8 DRG neurons.A**) Sample traces of voltage-activated outward currents in a TTXs/TRPM8 cell recorded at 24°C (left) and 10°C (middle). The right panel is the digital subtraction between the two temperatures, showing the cold-susceptible component of the voltage-activated outward currents. **B**) Summary of the experiments illustrated in A for TTXs/TRPM8 cells. **C**) Summary of the voltage-activated outward currents at 24°C and 10°C in TTXr/TRPM8 cells. Data are mean ± SEM.

We examined outward currents at 24°C and 10°C and determined how cooling temperatures affected these currents in TRPM8-expressing cells (Figure 4). Figure 4A shows an example of outward currents in a TTXs/TRPM8 cells at 24°C (Figure 4A left) and 10°C (Figure 4A middle). Outward currents elicited at 24°C were strong but were substantially inhibited at 10°C. The inhibition was more prominent at the beginning portion of the outward currents. Subtraction of the outward currents at 10°C from the currents at 24°C yielded slowly inactivating currents that resembled A-type K^+^ currents (Figure 4A right) [17]. At 24°C, the amplitudes of the outward currents at different voltage steps were comparable between TTXs/TRPM8 (n = 10) and TTXr/TRPM8 cells (n = 10) (Figure 4B and C). For both subtypes of TRPM8-expressing cells, the amplitude of the outward currents was also similar at the cooling temperature of 10°C. Thus, the cold-susceptible components (i.e. the differences between two temperatures) of the outward currents were also not different between the two subtypes of TRPM8-expressing cells (Figure 4B and C).

**Figure 5:**
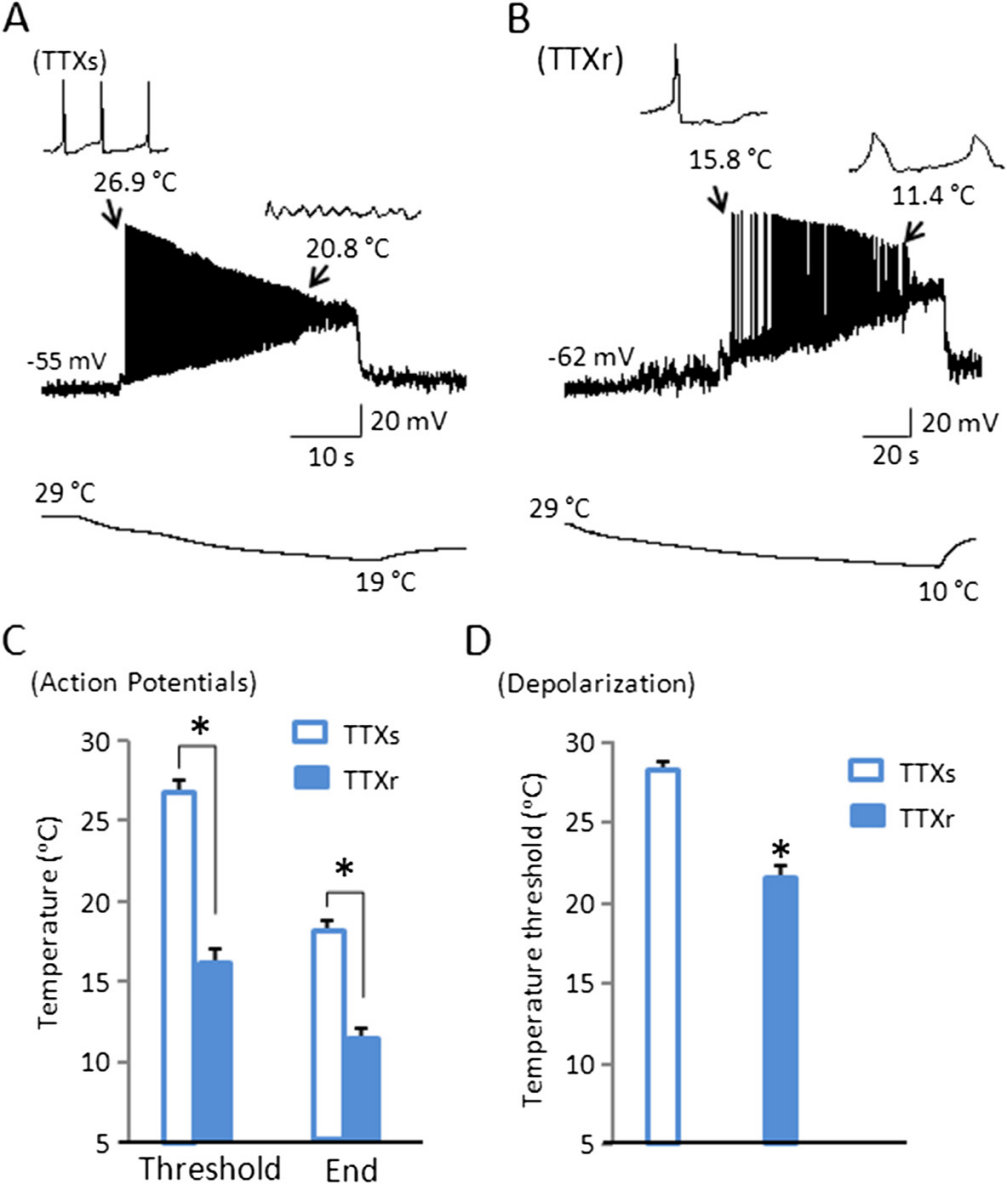
**Different temperature ranges of impulse generation between TTXs/TRPM8 and TTXr/TRPM8 DRG neurons.A-B**) Representative traces of action potential firing following a cooling temperature ramp in TTXs/TRPM8 neuron (A) and TTXr/TRPM8 neuron (B). Left insets in both (A) and (B) show threshold temperatures and initial action potentials (at expanded scale). Right insets show temperatures at which action potentials failed (A) or at which last one was recorded (B). The bottom traces show cooling temperature ramp from 29°C to 19°C for the TTXs cell (A) and from 29°C to 10°C for the TTXr cell. **C**) Summary data showing the threshold temperatures at which TTXs/TRPM8 (n = 9) and TTXr/TRPM8 cells (n = 7) started to fire action potentials and the temperatures at which action potentials failed or cooling was ended. (**D**) Summary data showing the threshold temperatures at which TTXs/TRPM8 (n = 9) and TTXr/TRPM8 cells (n = 7) started to depolarize in response to the cooling temperature ramp. Data represent mean ± SEM, *P < 0.05.

**Figure 6:**
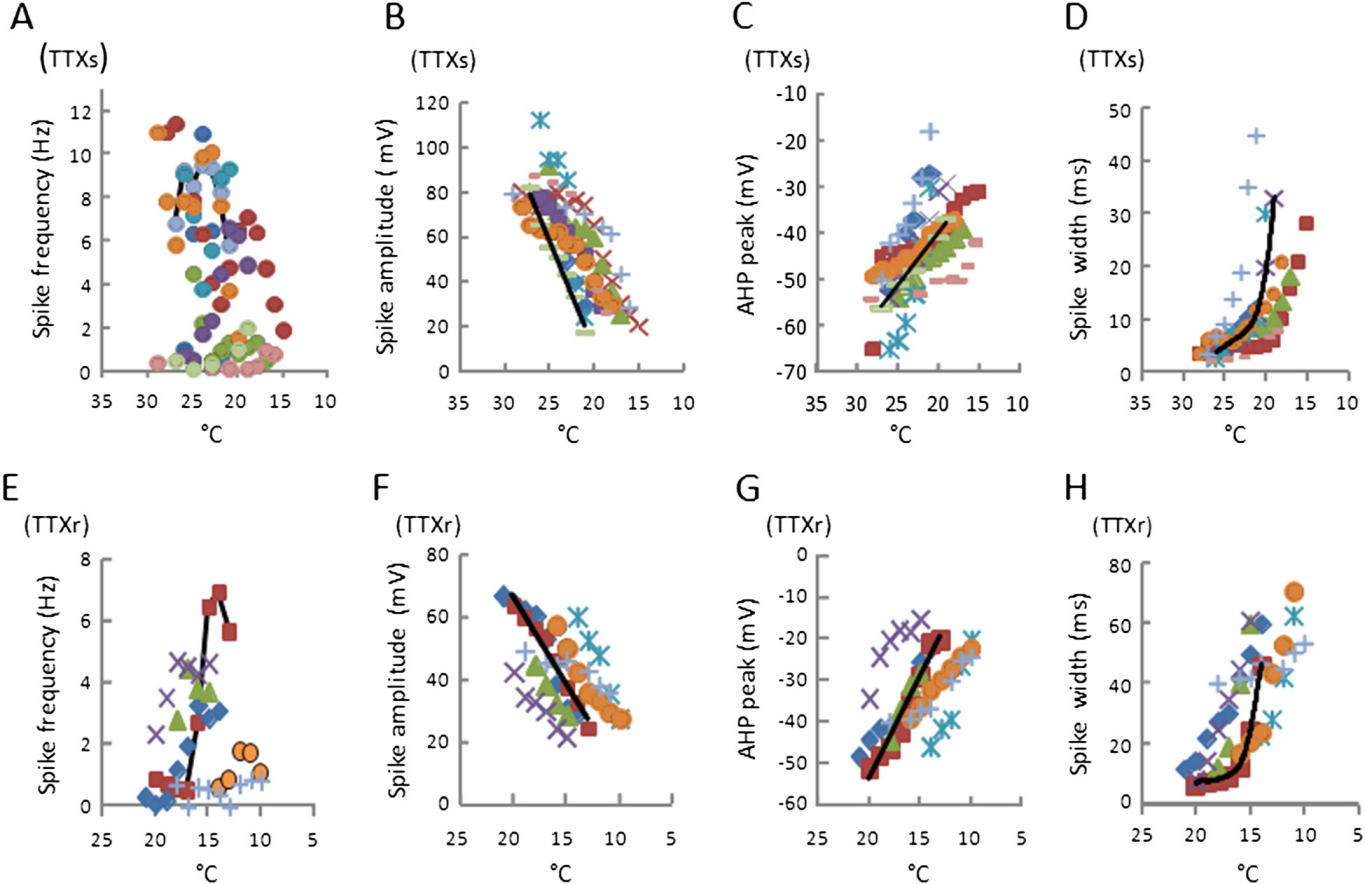
**Changes of membrane and action potential parameters during cooling in TTXs/TRPM8 and TTXr/TRPM8 DRG neurons. A-D**) Scatter plots of membrane and action potential parameters of 9 individuals TTXs/TRPM8 cells during cooling ramps. **E-H**) Scatter plots of membrane and action potential parameters of 7 individual TTXr/TRPM8 cells during cooling ramps. In each experiment, a slow ramp of cooling temperatures was started at 29°C. Different cells are shown in different colors (symbols). Black lines in each panel were best fit for each category from one TTXs/TRPM8 and one TTXr/TRPM8 cell chosen to best represent each group. Cold/menthol-sensitive cells were pre-identified with calcium imaging before current-clamp experiments. Scatter plots are used to present the changes of these parameters during cooling because the temperatures at which action potential firing started and ended were not exactly the same among these cells.

After switching recordings to current-clamp configuration, we examined action potential firing of TTXs/TRPM8 and TTXr/TRPM8 cells as they responded to slow ramps of cooling temperatures from 29°C (Figure 5). TTXs/TRPM8 cells responded to the cooling temperature ramp with membrane depolarization that started at 27.6 ± 0.4°C (n = 9, Figure 5D), and action potential firing started at 26.6 ± 0.7°C (n = 9, Figure 5A and C). Action potential firing adapted with a rapid reduction of action potential amplitudes as temperatures decreased, and action potentials were completely aborted at 18.3 ± 0.9°C (n = 9, Figure 5A). Spike frequency of TTXs/TRPM8 cells changed during cooling, but there was no consistent pattern in spike frequency over the cooling temperatures although there was a trend of reduction of spike frequency. Spike frequency was at its highest at 26°C with 6.0 ± 1.3 Hz (Figure 6A, n = 9). The reduction of spike amplitude followed linear decay with a slope of −8.0 ± 1.4 mV/°C in TTXs/TRPM8 cells (Figure 6B, n = 9,). The AHP peak of the first action potential was −55.6 ± 1.9 mV, and was reduced linearly during cooling with a slope of (−3.3 ± mV/°C, n = 9, Figure 6C). The width of the first action potential was 3.3 ± 0.2 ms (n = 9) and was increased exponentially during cooling with a constant of 2.35 ± 0.3 ms (Figure 6D, n = 9). Membrane depolarization increased during cooling, reaching a maximum membrane depolarization at the potential of −27.2 ± 2.5 mV (n = 9) before the final spike was seen. Thus, at temperatures where action potentials were aborted, membrane potential remained depolarized at values above action potential threshold, suggesting that the adaptation is not related to a possible TRPM8 desensitization.

In contrast to TTXs/TRPM8 cells, TTXr/TRPM8 cells responded to cooling temperatures with membrane depolarization at the temperature of 21.8 ± 0.5°C (n = 7, Figure 5D) and started to fire action potentials only when temperatures reduced to 17.9 ± 0.7°C (n = 7, Figure 5B and C). While some of these TTXr/TRPM8 cells stopped action potential firing before the end of cooling temperature of 10°C (n = 4), other cells continued all-or-none action potentials at 10°C (n = 3), the lowest temperatures used in this study. The average temperature for these cells to stop firing action potentials was 12 ± 0.4°C (n = 7, Figure 5C). Spike frequency increased during cooling with action potential frequency peaking at 3.4 ± 0.8 Hz (n = 7, Figure 6E) at 15°C. Action potentials displayed a reduction in amplitude with decreasing temperatures and the reduction of spike amplitude followed a linear decay with a slope of −5.1 ± 0.8 mV/°C (n = 7, Figure 6F). The threshold of first action potential was −11.2 ± 1.8 mV, significantly higher than the threshold of action potentials evoked by current injected at 24°C (−19.3 ± 2.0 mV, n = 11, P<0.05). The AHP peak of the first action potential was −41.8 ± 1.8 mV (n =7), and was further reduced linearly during cooling with a slope of −4.0 ± 0.6 mV/°C (Figure 6G). The width (W_1/2_) of the first action potential was broadened to 16.6 ± 4.4 ms (n = 7), and was further increased exponentially during cooling with a constant of 3.5 ± 0.9 ms/°C (n = 7, Figure 6H). The changes in both AHP peak and AP width were consistent with a significant inhibition of A-type K^+^ currents at cooling temperatures. Membrane depolarization increased during cooling, reaching a maximum membrane potential of −14.0 ± 2.3 mV (n = 7) before the final spike was seen.

## Discussion

In the present study, we showed that TTXs/TRPM8 cells fired action potentials in innocuous cooling temperatures ranging from 26.5°C to 18°C. This suggests that these cold-sensing neurons convey innocuous but not noxious cold stimuli. For TTXr/TRPM8 cells, we found that they fired action potentials in cold temperatures below 18°C, a temperature threshold below which noxious cold sensations could be generated in humans [1]. This result suggests that TTXr/TRPM8 cells represent a subpopulation of sensory neurons that convey noxious but not innocuous cold stimuli. Based on action potential properties shown in the present study as well as on our previous characterization of chemical sensitivities [16], TTXs/TRPM8 and TTXr/TRPM8 neurons were most likely to be non-nociceptive and nonciceptive neurons, respectively. Thus, our study demonstrated distinct sensory outputs of non-nociceptive and nociceptive TRPM8-expressing afferent neurons in response to innocuous and noxious cold stimuli, respectively. Our finding also highlighted that sensory discrimination between innocuous and noxious cold can be achieved with a single type of cold transducer.

Action potential firing induced by cooling temperatures in the present study was a result of TRPM8 channel activation, which led to membrane depolarization prior to action potential firing. The involvement of TRPM8 activation is supported by our observation that cooling temperatures evoked action potential firing in menthol-sensitive (Figure 5) but not in menthol-insensitive DRG neurons. When membrane depolarization rather than action potential firing was considered, our TTXs/TRPM8 cells had a temperature threshold of 27.6°C, consistent with previous studies of low threshold cold-sensing cells using a Ca^2+^ imaging method [22-24]. On the other hand, TTXr/TRPM8 neurons had a temperature threshold of 22°C for membrane depolarization, which is similar to the high threshold cold-sensing trigeminal neurons observed using a Ca^+^-imaging technique [22-24]. Our TTXr/TRPM8 cells were most likely to be polymodal nociceptors that detect noxious cold and other nociceptive stimuli as our previous study showed that these cells also expressed TRPV1 and P2X receptors [15,16]. This subpopulation of cold-sensing cells is similar to the nociceptors described by others [18,22]. While temperature thresholds based on membrane depolarization are useful indicators to differentiate two types of cold-sensing afferent neurons, it should be noted that temperature thresholds of action potential firing rather than those of cold-induced membrane depolarization are relevant to psychophysically-defined innocuous and noxious cold sensations because cold sensations cannot be formed by membrane depolarization without action potential firing. Indeed, the temperature thresholds of action potentials revealed in the present study are in good agreement with those defined psychophysically [1].

The temperature thresholds of action potential firing in TTXs/TRPM8 and TTXr/TRPM8 cells are determined by at least two factors, the expression level of functional TRPM8 and the voltage threshold of action potential firing. We have previously shown that TRPM8 expression was higher in TTXs cells than in TTXr cells [15,16]. Corroboratively, TRPM8-mediated responses were higher in low threshold-cold sensing cells than in high threshold-cold sensing cells [22]. For the second factor, the voltage threshold of the action potential was significantly lower in TTXs/TRPM8 than in TTXr/TRPM8 cells (Table 1). Combined, these two factors favor cold-induced action potential firing at higher temperatures in TTXs/TRPM8 cells compared with TTXr/TRPM8 cells.

We showed in the present study that voltage-activated inward currents, mainly carried by voltage-activated Na^+^ currents, were almost completely abolished in TTXs/cells but remained substantially large in TTXr/TRPM8 cells at 10°C. This result from cold-sensitive cells was in agreement with our previous study using cold-insensitive TTXs and TTXr cells [17]. The strong inhibition of TTXs Na^+^ currents has been attributed to the enhanced voltage-dependent slow inactivation of TTXs channels at cooling temperatures [17,18]. On the other hand, TTXr Na^+^ currents were not found to have significant voltage-dependent slow inactivation [17,18], and thereby TTXr currents remained substantially large at noxious cold temperatures. In cold-sensitive cells, we also showed that voltage-activated outward currents were significantly inhibited at cooling temperatures of 10°C for both TTXs/TRPM8 and TTXr/TRPM8 cells. The cold-susceptible components of the outward currents resembled A-type K^+^ currents. This result was consistent with our previous study in cold-insensitive DRG cells showing that A-type K^+^ channels but not non-inactivating K^+^ currents were significantly inhibited by cooling temperatures [17]. In TTXs/TRPM8 cells, the combination of cooling effects on TTXs channels and A-type K^+^ currents are likely to be the causes of action potential abortion near noxious cold and thereby set a temperature range for TTXs/TRPM8 cells to code innocuous cold only. On the other hand, in TTXr/TRPM8 cells, the substantial large TTXr currents that remained at low cooling temperatures would permit TTXr/TRPM8 cells to generate action potentials at nociceptive cooling temperatures. In addition, A-type K^+^ currents served as a brake to prevent high threshold cold-sensitive cells from being excited [22] and the inhibition of A-type K^+^ currents by noxious cold would release this brake to facilitate action potential firing in the noxious cold range. Thus, the thermal susceptibility of voltage-gated Na^+^ channels and A-type K^+^ channels, together with cold transducer TRPM8, set temperature ranges for sensory impulses conducted by both non-nociceptive and nocicpetive cold-sensing afferents, thus providing sensory discrimination between innocuous and noxious cold.

## Competing interests

The authors declare that they have no competing interests.

## Authors’ contributions

IS was involved in carrying out the electrophysiological experiments and the data analysis. JL was involved in the cell preparations. GX helped the data analysis and interpretation. JG conceived the study, participated in its design and coordination, and helped to draft the manuscript. All authors read and approved the final manuscript.
